# A40 THE ROLE OF HYPOXIA-INDUCIBLE FACTOR IN CELLULAR METABOLIC ADAPTATION UPON *GIARDIA* INFECTION

**DOI:** 10.1093/jcag/gwad061.040

**Published:** 2024-02-14

**Authors:** E R DeMichele, O Sosnowski, T Allain, A G Buret

**Affiliations:** Biological Sciences, University of Calgary, Calgary, AB, Canada; Biological Sciences, University of Calgary, Calgary, AB, Canada; Biological Sciences, University of Calgary, Calgary, AB, Canada; Biological Sciences, University of Calgary, Calgary, AB, Canada

## Abstract

**Background:**

Recent findings have highlighted the integral role of oxygen tension in gastrointestinal (GI) homeostasis. During hypoxia when oxygen supply is limited, mammalian cells utilize the Hypoxia-Inducible Factor (HIF) complex to activate genes that promote cell survival and alter glucose metabolism. The GI system exists in a state of physiologic hypoxia, subject to further alterations by pathogens. Although tissue and blood parasites are known to influence tissue oxygen tension, little is known regarding the modulation of hypoxia and HIF by enteric parasites.

**Aims:**

This project aims to characterize the effect of *Giardia duodenalis*, a common enteric protozoan that perturbs gastrointestinal function leading to diarrheal disease, on the cellular hypoxic response. We hypothesize that hypoxia-associated genes are activated upon *Giardia* infection, leading to an adaptive metabolic response with increased glycolytic flux to maintain cellular bioenergetic homeostasis.

**Methods:**

Caco-2 colonic epithelial cells were infected with *Giardia* isolate GS/M (MOI 10) for 1.5 or 4.5 hours to capture early and peak HIF activation, under normoxic (21% O_2_) or hypoxic (1%O_2_, STEMCELL hypoxia incubator) conditions. RNA was extracted for RT-qPCR to assess transcriptional changes of known HIF-target genes. Intracellular metabolite analysis via liquid-chromatography mass-spectrometry (hydrophilic-interaction chromatography method) was performed to assess HIF-mediated metabolic changes in uninfected and *Giardia*-infected cells (MOI 10) exposed to the hypoxia mimetic DMOG or the HIF inhibitor PX-478.

**Results:**

Under normoxic conditions, genes associated with compensatory cellular stress responses such as *VEGFA, ANKRD37, GADD45A,* and glycolysis-associated genes *HK2, and LDHA* are upregulated in *Giardia*-infected cells in a time-dependent manner (*p*ampersand:003C0.05). Under hypoxic conditions, fewer HIF-target genes are upregulated (*e.g.*, *VEGFA, GADD45A, PGK1*; *p*ampersand:003C0.05), suggesting *Giardia*-infected cells behave similarly to uninfected cells with constant HIF activation. Analysis of the Caco-2 intracellular metabolome indicates HIF-dependent changes in amino acid (e.g., glycine, threonine) and nucleic acid (e.g., guanosine, inosine, uracil) metabolism. DMOG treatment altered the production of glycolytic intermediates (e.g., DHAP, PEP), confirming the promotion of glycolytic flux by HIF.

**Conclusions:**

Both cell-stress-related and glucose-metabolism HIF target genes are activated upon *Giardia* infection. As well, some metabolic changes are dependent on HIF activation. These novel findings indicate *Giardia* promotes a hypoxic state in human intestinal cells, highlighting a metabolic cell rescue mechanism in response to enteropathogens. Furthermore, understanding the role of hypoxia in enteric parasitic infections will shed light on the potential of HIF as a therapeutic target.

**Funding Agencies:**

Natural Sciences and Engineering Research Council of Canada (NSERC)

